# NRP2 transcriptionally regulates its downstream effector WDFY1

**DOI:** 10.1038/srep23588

**Published:** 2016-03-30

**Authors:** Samikshan Dutta, Sohini Roy, Navatha S Polavaram, Gustavo B. Baretton, Michael H. Muders, Surinder Batra, Kaustubh Datta

**Affiliations:** 1 Department of Biochemistry and Molecular Biology, University of Nebraska Medical Center, Omaha, Nebraska, U.S.A; 2 Institute of Pathology, University Hospital Carl Gustav Carus, University of Technology of Dresden, Germany; 3 Fred & Pamela Buffett Cancer Center, Eppley Institute for Research in Cancer, Omaha, Nebraska, U.S.A

## Abstract

Neuropilins (NRPs) are cell surface glycoproteins that often act as co-receptors for plexins and VEGF family receptors. Neuropilin-2 (NRP2), a family member of NRPs, was shown to regulate autophagy and endocytic trafficking in cancer cells, a function distinctly different from its role as a co-receptor. WD Repeat and FYVE domain containing 1 (WDFY1)–protein acts downstream of NRP2 for this function. Our results indicated that NRP2 maintains an optimum concentration of WDFY1 by negatively regulating its expression. Since increased expression of WDFY1 reduces the endocytic activity, maintenance of WDFY1 level is crucial in metastatic cancer cells to sustain high endocytic activity, essential for promotion of oncogenic activation and cancer cell survival. Here, we have delineated the underlying molecular mechanism of WDFY1 synthesis by NRP2. Our results indicated that NRP2 inhibits WDFY1 transcription by preventing the nuclear localization of a transcription factor, Fetal ALZ50-reactive clone 1 (FAC1). Our finding is novel as transcriptional regulation of a gene by NRP2 axis has not been reported previously. Regulation of WDFY1 transcription by NRP2 axis is a critical event in maintaining metastatic phenotype in cancer cells. Thus, inhibiting NRP2 or hyper-activating WDFY1 can be an effective strategy to induce cell death in metastatic cancer.

Neuropilins (NRPs) are the cell surface glycoprotein receptors for class-3 semaphorins and vascular endothelial growth factors (VEGFs)^1^,^2^,^3^. There are two classes of NRPs namely, NRP1 and NRP2. Both the neuropilins have an extra-cellular N-terminal ligand binding domain, followed by a membrane insertion domain and a small C-terminal cytosolic tail, which lacks kinase activity^4^. Although there are sequence and structural similarities in the extracellular N-terminal domains of NRP1 and 2, the cytosolic tails are different^4^,^5^. Neuropilins often function as co-receptors for several cell surface receptors such as plexins, VEGF receptors, c-Met and contribute to various physiological and pathological conditions like neuronal development, angiogenesis, immunity, cancer cell survival and metastasis^5^,^6^,^7^,^8^,^9^. Neuropilins can also function without being a co-receptor^10^. NRP1 has been shown to regulate vascular sprouting formation as well as can induce angiogenesis independent of VEGFR2^11^,^12^. NRP1 with its PDZ domain at the C-terminal end of cytosolic tail binds GIPC molecules and thereby regulates the cellular cytoskeleton structure^13^.

We have previously reported a survival promoting function of NRP2 in cancer cells by enhancing autophagy during therapeutic stress^14^,^15^,^16^. In our recent study, we further showed that the expression of NRP2 can be a poor prognostic factor for invasive bladder cancer patients treated with radiochemotherapy^17^. NRP2 expression was associated with an increased risk of an early cancer specific death among these patients supporting its role as a promoter of therapy resistance^17^. It is therefore important to study NRP2 axis in cancer cells, where its function is distinct from its known role in inducing angiogenesis and neurogenesis.

WD40 repeats and single FYVE domain containing protein 1 (WDFY1) functions downstream of NRP2. It co-localizes with EEA1 positive early endosomes^18^,^19^ and acts as an adaptor molecule for protein-protein or protein-DNA interaction^18^,^20^,^21^ due to the presence of propeller-like structures made by several antiparallel beta-sheets. It acts as an adaptor protein for Toll like receptor 3/4 for the generation of inflammatory cytokines and type I interferons^18^,^22^. WDFY1 expression can be associated with mitochondrial dysfunction related to Alzheimer’s disease^23^. Reports have indicated a role of WDFY1 in maintaining hematopoietic stem cells^21^. Previously, we have reported that WDFY1 is the downstream effector of NRP2^15^,^16^. Our results indicated that the increased expression of WDFY1 following NRP2 depletion in cancer cells, leads to defective endocytic trafficking and autophagy^15^,^16^,^24^. Further, it was shown that over-expression of WDFY1 abrogated the early endosomal maturation, and thereby hindered the fusion of autophagosomes with the late endosomes for the formation of autolysosomes^24^.

In this study, we have delineated a novel mechanism for NRP2-mediated regulation of WDFY1 expression in cancer cells. Our results have shown that NRP2, by regulating the sub-cellular localization of a transcription repressor, fetal ALZ50-reactive clone 1 (FAC1), controls the transcription of WDFY1. As cancer cells depend on their endocytic activities to maintain the metastatic phenotype^25^, targeting NRP2/WDFY1 axis can be an effective therapeutic strategy for metastatic cancer.

## Results

### NRP2 axis regulates the expression of FYVE domain containing protein, WDFY1

**Figure 1:**
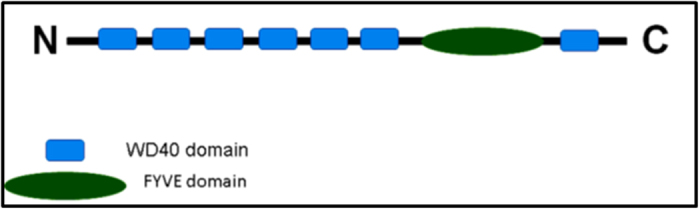
Domains arrangement of WDFY1 and WDFY2 proteins.

**Figure 2:**
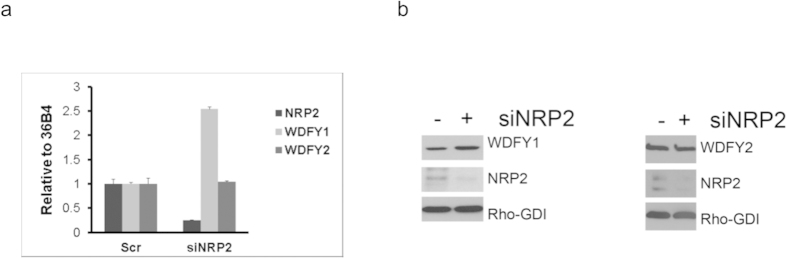
NRP2 controls WDFY1 expression. (**a**) mRNA expression of WDFY2 and WDFY1 following depletion of NRP2 in PC3 cells. (**b**) Immunoblot for WDFY2 and WDFY1 following depletion of NRP2 in PC3 cells.

Previously, we performed a microarray analysis to identify the downstream genes regulated by the VEGFC/NRP2 axis (Geo accession number GSE36085)^16^. Among the downstream genes, we were particularly interested in a FYVE domain containing protein WDFY1, because of its potential involvement in endocytic trafficking. Interestingly WDFY1 is the only FYVE domain containing protein, whose expression is similarly influenced following the depletion of NRP2 or VEGFC^15^,^16^. This was further confirmed when we tested the expression of two very similar FYVE domain containing proteins, WDFY1 and WDFY2. WDFY2 contains one FYVE and multiple WD40 domains similar to WDFY1 (Fig. 1) and is known to regulate early endosomal assembly^26^,^27^. Earlier reports including ours have indicated different cellular localization of WDFY1 and WDFY2. WDFY2 is predominantly associated with APPL1 positive vesicles^27^,^28^ and partially co-localize with EEA1 positive vesicles (Supplementary Fig. 1); whereas WDFY1 is present mainly in EEA1 positive vesicles. It therefore appears that WDFY1 functions downstream of WDFY2 for regulating the endocytic trafficking. We determined the mRNA and protein expression of these two molecules following depletion of NRP2 in prostate cancer cell line PC3 (Fig. 2a,b). Our results indicated that depletion of NRP2 though increased both the mRNA and protein level of WDFY1, had no effect on WDFY2 expression. These results therefore suggested that the regulation of WDFY1 expression by NRP2 is specific and is important for its function in regulating endocytic trafficking. It is therefore important to understand the underlying mechanism/s by which NRP2 regulates WDFY1 expression.

### Depletion of NRP2 does not alter WDFY1 mRNA and protein stability

**Figure 3:**
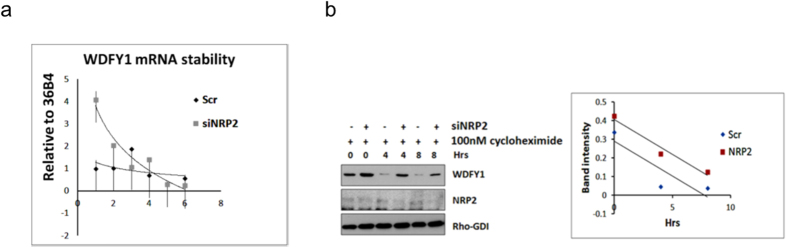
mRNA and protein stability of WDFY1 did not increase following NRP2 depletion. (**a**) WDFY1 mRNA stability in scr and NRP2 depleted PC3 cells following the treatment with translational inhibitor Actinomycin D. Real-time PCR was carried out for various time points following treatment and a line graph drawn to compare the stability between scrambled control and siNRP2 treated cells. (**b**) 50 mg/mL Cycloheximide was added to PC3 cells following NRP2 depletion for 48hrs and was chased for various time periods. WDFY1 protein stability was analyzed by western blot. Graph indicates the densitometric scanning of the immunoblot results following normalization with control Rho-GDI values.

We initially tested whether the depletion of NRP2 can enhance the stability of WDFY1 mRNA. We performed RNA stability assay with transcriptional inhibitor actinomycin D (Fig. 3a). Comparing the mRNA stability between the scrambled and siNRP2 treated PC3 cells following administration of the drug for various time points, we observed a decrease in WDFY1 mRNA stability in NRP2 depleted cells. Therefore, this result suggests that mRNA stability is not the primary factor for the NRP2 axis driven change in WDFY1 expression.

Next, the stability of the WDFY1 protein was measured after treating cells with a protein synthesis blocker (cycloheximide) at different time points in both NRP2-depleted and control cells (Fig. 3b). No difference in the rate of WDFY1 protein degradation was observed in scrambled and siNRP2-transfected PC3 cells. Both, mRNA and protein stability assays therefore suggested that NRP2 does not regulate WDFY1 expression by altering its mRNA or protein stability.

### Depletion of NRP2 increases WDFY1 promoter activity

**Figure 4:**
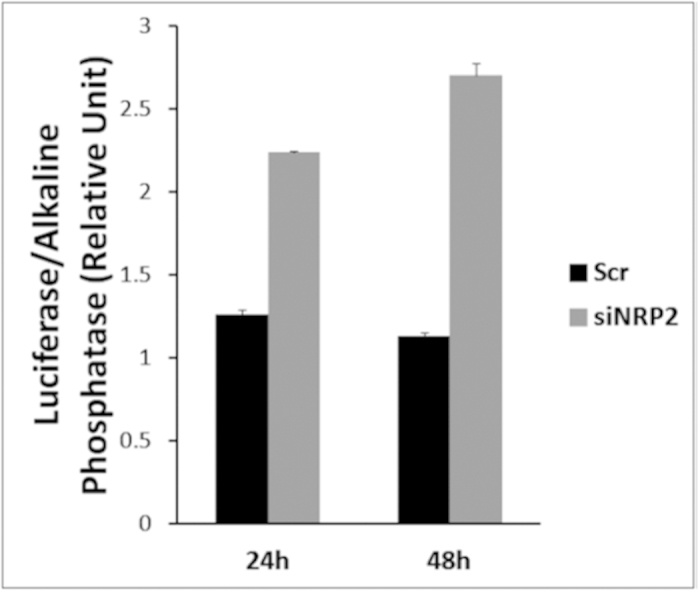
Depletion of NRP2 increased the WDFY1 promoter activity. Promoter activity of WDFY1 in scrambled and siNRP2 -treated PC3 cells. Culture media was used to assess the activity of secreted GLuc and was normalized with the activity of SEAP. Data are represented as a relative change in luciferase activity.

In order to test whether NRP2 can regulate *WDFY1* transcription, we performed promoter activity assay of *WDFY1* (Fig. 4). The promoter construct was generated by inserting 1490bp upstream of the transcriptional start site of *WDFY1* into pEZX vector containing the Gaussia Luciferase reporter gene. WDFY1 promoter construct was then transfected into PC3 cells followed by treatment with either scramble or NRP2 siRNA as described in the materials and methods section. Following NRP2 depletion, a significant increase in the transcriptional activity of *WDFY1* was detected (Fig. 4). This result therefore suggested that the enhanced expression of *WDFY1* following knocking down of *NRP2* is due to the increased transcriptional activity of *WDFY1*.

### NRP2 regulates the function of transcriptional repressor, FAC1, for WDFY1 transcription

**Figure 5:**
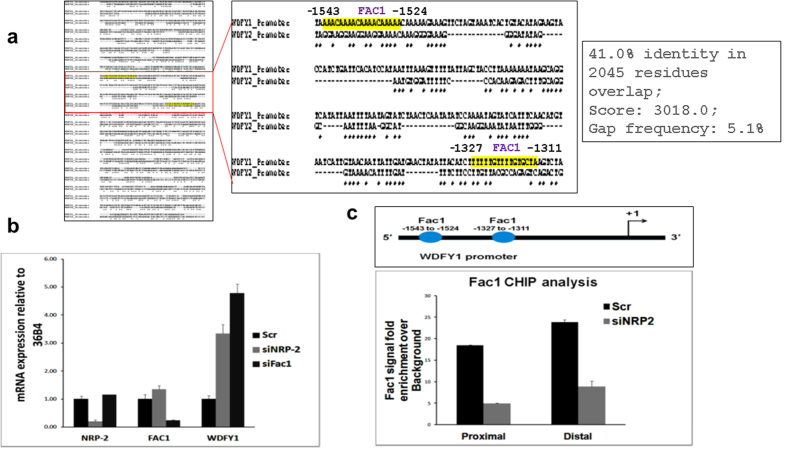
Fac1 controls WDFY1 transcription. (**a**) Comparing the promoter sequence of WDFY1 and WDFY2. The red boxed region was amplified and the magnified image shown in right. The yellow highlighted region indicates the FAC1 binding sites in WDFY1 promoter. (**b**) Quantification of relative expression of WDFY1 mRNA using RT-PCR following the depletion of NRP2 and FAC1. (**c**) CHIP analysis showing the recruitment of FAC1 to the WDFY1 promoter region following depletion of NRP2. Data are represented as a change in signal intensities over the background. Rabbit IgG was used as an internal control.

To understand the potential transcriptional factors binding sites within the WDFY1 promoter, we took an *in silico* approach for mapping the promoter region. Using the software from BioBase (Qiagen) with the stringent cut-off setup to predict the possible transcription factors, we found a list of several potential transcriptional regulators within the 2000bp WDFY1 promoter (Table 1). WDFY2 gene, which is structurally and functionally similar to WDFY1^26^ but not under the regulation of the NRP2 axis, also shares comparative promoter sequence (Fig. 5a). Sequence analysis has revealed that there is almost 41% homology between the promoter region of WDFY1 and WDFY2 genes (Fig. 5a). Further, using the same threshold value of the BioBase software, we analyzed the prospective transcription factors recognition sites within the promoter region of WDFY2 gene. To understand how NRP2 regulates the transcription of WDFY1, we ruled out the transcriptional regulators which are common within the WDFY1 and WDFY2 promoter regions (Table 1). Among the specific sets of regulators that are present only in WDFY1 promoter, we selected those that have multiple binding sites. Of specific interest among these factors was Fetal Alz-50 Clone 1 (FAC1), which is a known transcriptional repressor^29^,^30^,^31^. The FAC1 protein contains several domains such as a plant homeodomain/leukemia associated protein (PHD/LAP) zinc finger motif, an acidic domain, a PEST sequence, and nuclear localization sequences classically present in transcription regulators^30^,^31^. Interestingly, loss of FAC1 activity has been shown in Alzheimer’s disease^31^. Also, over-expression of WDFY1 is also associated with the senile plaque formation in Alzheimer’s disease. The study herein demonstrated several FAC1-binding sites both in the proximal and distal regions of the WDFY1 promoter region. Thus, we can postulate that the FAC1 protein is a transcriptional repressor of WDFY1, and the binding of FAC1 to WDFY1 promoter regions is regulated by the NRP2 axis. Interestingly, depletion of FAC1 increased the level of WDFY1 mRNA (Fig. 5b) indicating FAC1 is the negative regulator of WDFY1 transcription.

### NRP2 regulates the FAC1 binding within WDFY1 promoter

We performed CHIP assay to determine whether FAC1 is recruited to the *WDFY1* promoter. There are two potential binding sites for FAC1 within the *WDFY1* promoter region. The CHIP assay with a FAC1-specific antibody showed that FAC1 was recruited to its predicted binding sites located at both the proximal and distal regions of the *WDFY1* promoter. To confirm that FAC1 recruitment to the *WDFY1* promoter is influenced by NRP2, we tested FAC1 binding to *WDFY1* promoter following the knocking down of *NRP2*. Our results indicated that NRP2 depletion significantly reduced recruitment of FAC1 to both the proximal and distal binding sites within the *WDFY1* promoter (Fig. 5c). These results therefore suggested that NRP2 axis regulates the recruitment of FAC1 to the promoter regions of *WDFY1* and thereby down-regulates the transcriptional activity of *WDFY1*. Based on our extensive review of the related literature, no published report exists to date that addresses how WDFY1 expression is regulated in the cell. Therefore, we are the first to delineate the signaling event that regulates expression of the *WDFY1* gene.

**Figure 6:**
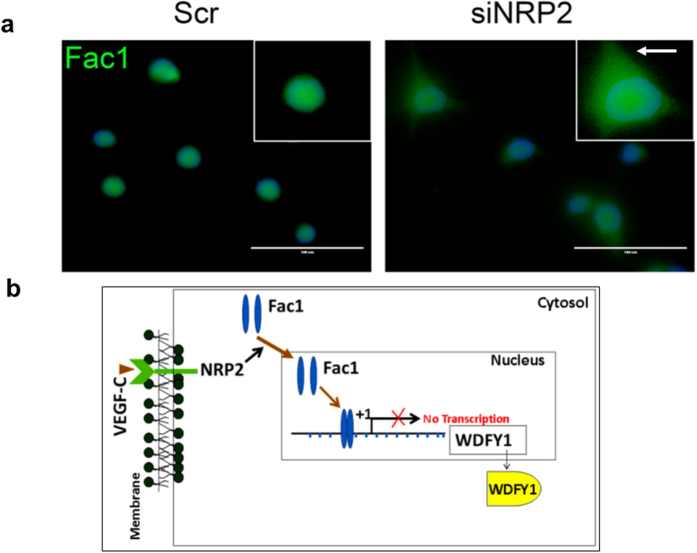
NRP2 controls Fac1 sub-cellular localization. (**a**) Change in Fac1 sub-cellular localization following NRP2 depletion in PC3 cells. Nucleus was stained with DAPI. Arrow indicates the cytosolic region. Scale Bar indicates 100 μm. (**b**) Schematic diagram showing the regulation of WDFY1 by NRP2 axis.

Next, we would like to answer how FAC1 is regulated by the NRP2 axis. Our immunostaining data indicated that at steady state, FAC1 is present both in cytosol as well as within the nucleus of the prostate cancer cells. However, upon depletion of the NRP2 axis, there is a shift in FAC1 localization from nucleus to cytosol (Fig. 6a). Our result therefore indicates that upon depletion of NRP2, FAC1 is removed from the *WDFY1* promoter and preferentially moves towards the cytosolic region of the cell, thereby releasing the inhibition of *WDFY1* transcription.

## Discussion

Our recent reports showed a novel function of NRP2 in the maturation of early to late endosomes, necessary for the maintenance of cell surface receptor activation during metastatic progression and autophagic activity during therapeutic stress^16^,^24^. WDFY1 was identified as the immediate downstream target of NRP2 axis. In this paper we have provided the mechanism by which NRP2 regulates WDFY1 expression (Fig. 6b). Our results indicated that NRP2 regulates the transcription of WDFY1. We were also successful in identifying the transcriptional repressor of WDFY1, whose function is regulated by NRP2. All these information would be important for targeting the NRP2 axis in treating metastatic cancer.

Not much is known about FAC1 dependent transcriptional regulation in cancer cells. However it has been shown that FAC1 plays an important role in regulating the expression of genes associated with various neurodegenerative diseases and has been identified within the amyloid plaques of Alzheimer’s patients^29^,^30^,^31^,^32^,^33^,^34^. FAC1 usually functions as a transcriptional repressor. It itself is transcribed as a smaller transcript of the gene called *bromodomain plant homeodomain transcription factor* (*BPTF*)^32^. FAC1 shares the N-terminal sequence homology with its parental protein BPTF and comprises the consensus DNA binding motif^29^,^32^. However, FAC1 lacks the C-terminal bromodomain of BPTF. Previous reports indicated that the subcellular localization of FAC1 is important in regulating the embryonic as well as neuronal development^32^. Here we showed that in prostate cancer cells, FAC1 was directly recruited to the WDFY1 promoter and inhibited its transcription in the presence of NRP2. Depletion of NRP2 favored the cytoplasmic retention of FAC1 and thereby facilitated WDFY1 transcription. Importantly, the subcellular localization of FAC1 is often regulated through its phosphorylation. It is currently unknown whether NRP2 controls the phosphorylation status of FAC1. Lack of phosphorylation domain specific antibody for FAC1 has made it difficult to characterize the endogenous phosphorylation status of FAC1 in the presence or absence of NRP2. Since our result (Fig. 5b) suggested no change in FAC1 protein level in cells upon knocking down *NRP2*, we predict post-translational modification of FAC1 by NRP2 is the possible cause of NRP2 mediated cellular localization of FAC1.

In summary, we observed a novel transcriptional regulation of WDFY1 by NRP2. This function is important in maintaining a high endocytic activity in metastatic cancer cells as previously reported^25^,^35^,^36^. The endocytic activity in metastatic cancer cells not only favors the oncogenic activation of several cell surface receptors but also promotes processes such as autophagy to resist therapeutic stress. The NRP2/WDFY1 axis may regulate cellular functions beyond cancer cells. Increased WDFY1 expression is also responsible for manifestation of Alzheimer’s disease. Since neuronal cells often express NRP2, it would be interesting to determine if similar NRP2/WDFY1 axis can also function in neuronal cells.

## Materials and Methods

### Cell culture, Plasmid and transfection

The prostate cancer cell line PC3 was grown at 37 °C in RPMI media supplemented with 10% FBS as reported previously^16^,^24^.

WDFY1 promoter plasmid was purchased from Genecopoeia (HPRM11136). WDFY2-GFP plasmid was purchased from Origene (RG204225). On-target plus smart pool siNRNA for NRP2, FAC1 were purchased from Dharmacon (GE Dharmacon). Transfection of plasmids was carried out using Effectene reagent (Qiagene) and siRNA was transfected using DharmaFECT 2/3 (GE Dharmacon) as per the manufacturer’s protocol.

### mRNA isolation and qRT-PCR

RNeasy mini kit was used to isolate the total RNA from the cells following manufacturer’s protocol (Qiagen, 74104). cDNA was prepared from 1μg of mRNA using Transcriptor First Strand Synthesis Kit (Roche, Indianapolis, IN, 04379012001). Quantitative real-time PCR (qRT-PCR) was carried out with Power SYBR^®^Green master mix (Life Technologies, Grand island, NY, 4368706) as per our previously published protocol^16^.

### mRNA and Protein stability assay

PC3 cells were maintained in culture for 36hrs following *NRP2* depletion with siRNA. After 36hrs, 5 μg/ml Actinomycin D (Sigma A9415) was added to the cultured cells (in proliferating phase) and incubated at 37 °C for 2, 4 and 6 hrs. At the end of incubation, mRNA was isolated and cDNA prepared following the protocol mentioned above. Real time PCR was performed with WDFY1-specific primers. The housekeeping gene, 36B4 was used as an internal control.

For analysis of protein stability, cycloheximide assay was performed. Cells were grown for 48hrs following NRP2 depletion. Cycloheximide (50 μg/ml) (Sigma, C7698) was added into the cells for various time periods. Cells were harvested and protein isolated for immunoblot analysis.

### WDFY1 Promoter assay

WDFY1 promoter plasmid (1.49 kb) was purchased from Genecopoeia (HPRM11136), which contained Gaussia Luciferase (GLuc) and secreted Alkaline Phosphatase (SEAP) expressing genes as dual reporters. The promoter region was selected immediate upstream of the transcription start site and was placed next to *GLuc* reporter gene. *SEAP* gene expression was under the control of CMV promoter and therefore SEAP activity was measured to normalize any variation in transfection efficiency. NRP2 or scrambled siRNA was transfected into the cells after 6 hrs following the promoter plasmid transfection. Conditioned media in each experimental condition was collected 48 hours after the siRNA transfection. Luciferase and SEAP activity in the conditioned media were measured using Luminometer and results were displayed as ratios of GLuc and SEAP luminescence intensities.

### Chromatin immuno-precipitation (CHIP) assay

CHIP was performed using the MAGnify™ Chromatin Immunoprecipitation System (life technology, 492024). Cells were fixed with 1% formaldehyde for 10mins at room temperature followed by neutralization with glycine. Before lysis, cells were thoroughly washed with PBS. Cells (~1 × 10^6^) were lysed with lysis buffer using the Bioruptor until the chromatin fragment size was below 500bp. Chromatin fragments were immunoprecipitated with antibody-Dynabeads complex for 2 hrs at 4 °C. After vigorous washing with wash buffer for 5 times, reverse cross-linking was carried out at 55 °C for 15 min. DNA was eluted with the DNA purification magnetic beads supplied with the kit. Rabbit IgG was used as a negative control for the reaction. DNA was also isolated directly from the fragmented chromatin sample without performing the immunoprecipitation, which was used as a positive control and data normalization for qPCR. Amplification efficiency of the samples was derived from the slope calculated from the standard curve with 10-fold serial dilution of input Control DNA. Fold enrichment was calculated with the signals of each test reaction divided by the signals with negative control.

qPCR was performed using the WDFY1-promoter specific primers, which amplified the Fac1 binding regions. The primer sequences are as follows

Fac1_distal (-1524) Forward: 5′-CTGGGTGACGGAGTGAGTTC-3′

Fac1_distal (-1524) Reverse: 5′-CTTTTTGTTTTTGTTTTGTTTTGTT-3′

Fac1_proximal (-1327) Forward: 5′-TCATTTCAACATGTAATCATTGTAAC-3′

Fac1_proximal (-1327) Reverse: 5′-TTTTATAGACTTAGCACAAAACAAAAA-3′

## Additional Information

**How to cite this article**: Dutta, S. *et al.* NRP2 transcriptionally regulates its downstream effector WDFY1. *Sci. Rep.* **6**, 23588; doi: 10.1038/srep23588 (2016).

## Supplementary Material

Supplementary Information

## Figures and Tables

**Table 1:** Comparing the possible transcription factor binding site in WDFY1 and WDFY2 promoter region.

**WDFY1 WDFY2**	**WDFY1 WDFY2**
ZNF333*	YY1*
ZNF333*	AHR
CRX	NF-AT1
SRY*	ZNF333*
SOX10*	SREBP
CRX	DRI1*
HMGIY	HNF1
Pit-1	DRI1*
Pit-1	SOX10*
SOX10*	Xvent-1*
ING4*	myogenin*
Freac-3	myogenin*
ING4*	TATA*
SRY*	CDX-2*
**FAC1**	Pbx
**FAC1**	CPBP*
SRY*	Churchill*
SRY*	Muscle initiator
SRY*	Nanog
HSF1	NF-AT1
Gfi1	SRY*
ZNF333*	SOX10*
ER-alpha	NF-AT1
DRI1*	RBP-Jkappa
SOX10*	ZNF333*
DMRT4	MAF
Zfp105	MZF1
ZNF333*	GKLF
FAC1	SRY*
FAC1	LXR, PXR, CAR, COUP, RAR
SRY*	CREB1
TATA*	myogenin*
CDX-2*	myogenin*
Nkx-2.5	CPBP*
Tbx5	CPBP*
Tbx5	GEN_INI
ZNF333*	p53 decamer
CPBP*	NF-1
ING4*	BEN
Xvent-1*	LXR, PXR, CAR, COUP, RAR
ZNF333*	CPBP*
ER-alpha	SOX10*
ZNF333*	ZF5
GATA	p53
Bbx	p53
Bbx	Sp1
ZNF333*	MAZ
ZNF333*	CPBP*
DRI1*	CPBP*
ZNF333*	GKLF
HNF-3beta	BEN
myogenin*	MAZ
myogenin*	YY1*
SRY*	GEN_INI
YY1*	ZF5
ZNF333*	GKLF
SRY*	Egr-1
SOX10*	ING4*
MRF2	BEN
CPBP*	CPBP*
Churchill*	CPBP*
	Sp1
	CPBP*
	Churchill*
	Egr-1
	CPBP*

^*^indicates the common transcription factors between WDFY1 and WDFY2 promoter site.
