# Supplementary material for: NRP2 transcriptionally regulates its downstream effector WDFY1
Source: Sci Rep. 2016 Mar 30;6:23588. doi: 10.1038/srep23588 (PMC4812299; doi:10.1038/srep23588)
Supplement: Supplementary Information [file srep23588-s1.docx]

**NRP2 transcriptionally regulates its downstream effector WDFY1**

**Samikshan Dutta^1,§^, Sohini Roy^1^, Navatha S Polavaram^1^, Gustavo B. Baretton^2^, Michael H. Muders^1,2^, Surinder Batra^1,3^,Kaustubh Datta^1,3,§^**

^1^Department of Biochemistry and Molecular Biology, University of Nebraska Medical Center, Omaha, Nebraska

^2^Institute of Pathology, University Hospital Carl Gustav Carus, University of Technology of Dresden

### ^3^Fred & Pamela Buffett Cancer Center, Eppley Institute for Research in Cancer, Omaha, Nebraska

**Running Title:** NRP2 Regulates WDFY1 transcription

^§^**Corresponding Author:**

1. Kaustubh Datta, Ph.D.

Department Biochemistry and Molecular Biology, University of Nebraska Medical Center, Durham Research Center II, Room 4022

985870 Nebraska Medical Center

Omaha, NE 68198-5870

Tel: (402) 559-7404

Fax: (402) 559-6650

Email: [kaustubh.datta@unmc.edu](mailto:kaustubh.datta@unmc.edu)

1. Samikshan Dutta, Ph.D

Department Biochemistry and Molecular Biology, University of Nebraska Medical Center, Durham Research Center II, Room 4022

985870 Nebraska Medical Center

Omaha, NE 68198-5870

Tel: (402) 559-7404

Fax: (402) 559-6650

Email: [samikshan.dutta@unmc.edu](mailto:samikshan.dutta@unmc.edu)

**Keywords:** WDFY1, NRP2, FAC1,

**Conflicts of Interest:** The authors have no conflicts of interest to declare.

**Abstract**

**Supplementary Fig 1. WDFY2 partially co-localizes with EEA1 positive vesicles:** Immunostaining of WDFY2 (Green) and EEA1 (Red) following depletion of NRP2 and over-expressing WDFY2-GFP plasmid in PC3 cells. Nucleus was stained with DAPI. Scale Bar indicates 20µm.

Supplementary materials and methods:

Confocal Microscopy: PC3 cell were grown on poly-D-lysine coated coverslips. After 48hrs of transfection, cells were fixed with 4% para-formaldehyde followed by blocking (1% BSA in PBS and 0.5% saponin) for 1 hour. Next, cells were incubated with respective 1º antibodies for overnight at 4ºC followed by secondary antibodies for 1 hour. Zeiss 710 confocal microscopy was used for capturing the images.
